# Real-World Effectiveness and Safety of Belantamab Mafodotin Monotherapy in Triple-Class Refractory Multiple Myeloma

**DOI:** 10.3390/ijms241411829

**Published:** 2023-07-23

**Authors:** Ioannis Ntanasis-Stathopoulos, Panagiotis Malandrakis, Despina Fotiou, Magdalini Migkou, Foteini Theodorakakou, Maria Roussou, Evangelos Eleutherakis-Papaiakovou, Vassiliki Spiliopoulou, Efstathios Kastritis, Evangelos Terpos, Meletios-Athanasios Dimopoulos, Maria Gavriatopoulou

**Affiliations:** Department of Clinical Therapeutics, School of Medicine, National and Kapodistrian University of Athens, 157 72 Athens, Greece

**Keywords:** multiple myeloma, refractory, relapsed, belantamab mafodotin, effectiveness, safety, response, toxicity, cornea, monoclonal antibody, conjugated

## Abstract

B-cell maturation antigen (BCMA) is a promising therapeutic target for multiple myeloma (MM). The aim of this study was to assess the effectiveness and tolerability of monotherapy with the conjugated anti-BCMA monoclonal antibody belantamab mafodotin in triple-class refractory patients with MM in real-world practice. Patients refractory to at least one proteasome inhibitor, one immunomodulatory drug, and one anti-CD38 monoclonal antibody received belantamab mafodotin at 2.5 mg/kg intravenously every 3 weeks. Overall, 27 patients with a median age of 65 years (range 41–81) were included. Of these, 52% were male and the median number of prior lines of treatment was 5 (4–10). The overall response rate (partial response or better) was 52%, whereas the disease control rate (stable disease or better) was 70%. The median progression-free survival (PFS) was 2 months (95%CI: 0–7), whereas the median PFS among the responders was 12 months (95%CI: 6–18). Regarding the toxicity profile, the most common toxicity was eye toxicity, in 44% of the patients. Keratopathy grade 2–3 was reported in 33.3% of the patients. In conclusion, belantamab mafodotin showed a safety and efficacy profile consistent with the results of the registrational study. Importantly, heavily pretreated patients who responded to treatment derived a substantial survival benefit.

## 1. Introduction

Despite continuous improvement in survival, patients with multiple myeloma (MM) still relapse, and survival after failure of proteasome inhibitor (PIs), immunomodulatory drugs (IMiDs), and anti-CD38 monoclonal antibodies remains poor. Therefore, there is a need for new treatment strategies in these triple-class refractory patients. B-cell maturation antigen (BCMA) is a promising therapeutic target, as it is an antigen expressed specifically on plasma cells, with a higher expression in myeloma cells [1]. BCMA promotes myeloma cell growth, chemoresistance, and immunosuppression in the bone marrow microenvironment [1,2,3]. The expression of BCMA increases as the disease progresses from MGUS to advanced myeloma, whereas increased BCMA levels have been correlated with adverse prognosis [4].

Belantamab mafodotin belongs to a class of drugs called antibody–drug conjugates (ADCs). Belantamab mafodotin consists of an afucosylated IgG1 monoclonal antibody that specifically targets BCMA expressed on the surface of myeloma cells coupled with a potent cytotoxic agent named monomethyl auristin-F (MMAF). This unique combination allows for targeted delivery of the drug directly to the myeloma cells, minimizing on-target off-tumor damage to healthy tissues. By binding to BCMA and delivering the cytotoxic payload, belantamab mafodotin disrupts the growth and survival of myeloma cells, inhibiting tumor progression. It has shown promising results in clinical trials, demonstrating efficacy in patients for whom multiple lines of prior therapy have failed [5,6].

Belantamab mafodotin has been approved for patients with triple-class refractory relapsed/refractory multiple myeloma (RRMM) who have received at least four prior lines of treatment following the encouraging results from the DREAMM-2 study [5,6]. The DREAMM-2 clinical trial evaluated the efficacy and safety of belantamab mafodotin in patients with relapsed or refractory multiple myeloma who had received at least three prior lines of therapy, including an IMiD, a PI, and an anti-CD38 monoclonal antibody. The study reported an overall response rate (ORR) of 32%, with 19% achieving a very good partial response or better. The presence of extramedullary myeloma disease was an adverse predictive factor for response to treatment. The median duration of response (DOR) was 11 months, indicating that patients who responded to treatment experienced a sustained period of disease control. The median progression-free survival (PFS) for the whole study population was 2.8 months, reflecting a modest improvement in disease control compared to the previous lines of therapy these patients had received. Interestingly, the median overall survival (OS) was 13.7 months, suggesting a survival benefit in patients heavily pretreated with RRMM. Regarding the toxicity profile of belantamab mafodotin, the most commonly reported adverse events in the DREAMM-2 study included keratopathy (corneal epithelial changes), thrombocytopenia, anemia, and fatigue. However, most of these adverse events were manageable and reversible [5,6].

However, belantamab mafodotin is not currently commercially available from the Food and Drug Administration (FDA) in the United States due to the negative primary results of the phase 3 DREAMM-3 clinical trial comparing belantamab mafodotin to pomalidomide. Belantamab mafodotin did not reach the threshold of superiority to pomalidomide/dexamethasone in terms of median PFS prolongation (11.2 versus 7 months, respectively, *p* = 0.56), although the responses were more durable with belantamab mafodotin [7]. Furthermore, not all patients recruited in the DREAMM-3 study were triple-class refractory. Belantamab mafodotin remains an option for heavily pretreated patients with RRMM in Europe, whereas several studies are ongoing with novel combinations that are based on belantamab mafodotin both in the upfront and in the relapsed/refractory setting [8].

Real-world studies have a higher external validity than clinical trials, since they are deprived of the strict inclusion and exclusion criteria of the latter, and, thus, the real-world study population is more representative of clinical practice [9,10]. The robustness of clinical trial results based on strict trial design is necessary from a regulatory perspective; however, the extrapolation of clinical trial outcomes and the incorporation of the novel agents into everyday practice can be evaluated with real-world evidence [10,11,12]. In this context, the aim of the present study was to evaluate the effectiveness and tolerability of monotherapy with belantamab mafodotin in triple-class refractory MM patients in real-world practice.

## 2. Results

Overall, 27 patients with triple-class refractory MM who were treated with belantamab mafodotin were included in this analysis. The patients had a median age of 65 years old (range 41–81), 52% were male, and the median number of prior lines of treatment was five (4–10). Regarding the previous lines of treatment, all patients had received lenalidomide, bortezomib, and anti-CD38 treatment, whereas 70% had received pomalidomide and 89% had received carfilzomib. All patients were refractory to lenalidomide and anti-CD38 monoclonal antibody, whereas 14 (52%) were refractory to bortezomib, 24 (89%) were refractory to carfilzomib, and 19 (70%) were refractory to pomalidomide. No patient had previously received any anti-BCMA targeting agent. At the time of belantamab mafodotin initiation, 59% of the patients had abnormal LDH levels, and 19% of the patients were ISS 3 and 14% RISS 3 at diagnosis. Table 1 summarizes the baseline patient characteristics.

The overall response rate (ORR, PR, or better) in this cohort was 52%, whereas the disease control rate (DCR, SD, or better) was 70.4%. At best response, three patients (11.1%) had achieved complete remission (CR), five patients (18.5%) had very good partial response (VGPR), six patients (22.2%) had partial response (PR), one patient (3.7%) had a minor response (MR), four patients (14.8%) had stable disease (SD), and eight patients (29.6%) had disease progression. Only one out of three patients with EMP showed response.

One patient is still receiving therapy, whereas the median number of completed cycles of treatment for the whole cohort was 3 (range 1–32). The median number of lines of therapy post-treatment with belantamab mafodotin is 1 (range 0–8), and seven patients (26%) are currently alive. The cause of death for the 20 patients was myeloma progression in 16 (80%), infections in 3 (15%), and a fatal accident in 1. Four patients died while receiving treatment with belantamab mafodotin, two of them due to rapid disease deterioration, one had the fatal accident, and one died due to coronavirus disease (COVID-19).

**Figure 1:**
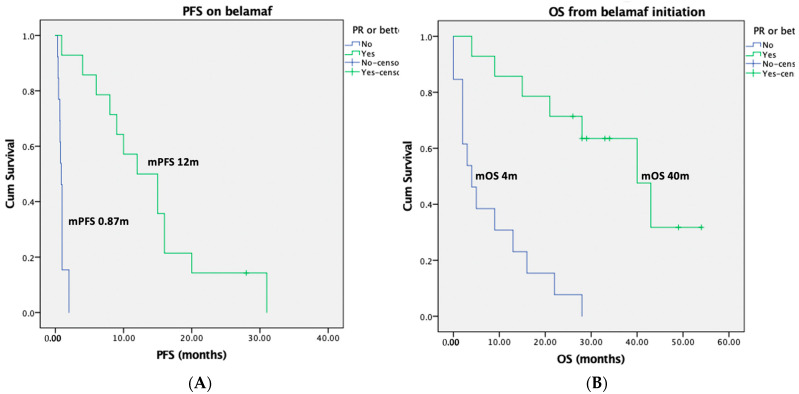
Kaplan–Meier curves of PFS (**A**) and landmark OS (**B**) from belantamab mafodotin (belamaf) initiation among responders (blue curves) and non-responders (green curves).

The median progression-free survival (PFS) for the entire group was 2 months (95% confidence intervals (CI): 0–7 months), whereas the median PFS among the responders was 12 months (95% CI: 6–18 months). The median PFS for non-responders was 0.87 months (95% CI: 0.69–1.05 months), log rank *p* < 0.001 for responders versus non-responders (Figure 1). Interestingly, the median PFS was 10 months (95% CI: 0–22 months) for patients who started belantamab mafodotin due to biochemical relapse, as compared to a median PFS of only 0.9 months (95% CI: 0.5–1.2 months) for patients with symptomatic myeloma relapse (log rank *p* = 0.001).

The median overall survival (OS) was 89 months (95% CI: 48–129 months) from MM diagnosis, whereas the landmark median OS was 16 months (95% CI: 2–30 months) from belantamab mafodotin initiation. The landmark median OS for the responders was 40 months (95% CI: 24–56 months), whereas the landmark median OS was only 4 months (95% CI: 0.5–8 months) for the non-responders (log rank *p* < 0.001, Figure 1). Interestingly, the median OS was 28 months (95% CI: 5–51 months) for patients who started belantamab mafodotin due to biochemical relapse, in comparison with a median OS of 4 months (95% CI: 0.8–7 months) for patients with symptomatic myeloma relapse (log rank *p* = 0.003).

Regarding the toxicity profile, the most common toxicity was eye toxicity, which was reported in 12 patients (44%). In nine patients, the eye toxicity was completely resolved, and in three patients, the eye-related adverse event was resolved with sequelae. More specifically, keratopathy grade 2–4 was reported in nine patients (33.3%); six patients had grade 2, two patients had grade 3, and one patient had grade 4. Keratopathy was resolved in all patients. However, one patient had to discontinue treatment due to a grade 4 corneal ulcer. Another patient discontinued treatment due to persistent grade 2 eye toxicity with slow resolution and the patient’s wish. A reduction in visual acuity grade 1–3 was reported in seven patients (26%), most of which were grade 2 (*n* = 5). One patient experienced two incidences of conjunctiva hemorrhage grade 2, and four patients had xeropthalmia (two grade 2 and two grade 1). Overall, dose reductions were necessary in eight patients (30%) due to ocular toxicity. Dose delays were reported in all patients with ocular toxicity. Upon documentation of corneal toxicity from the ophthalmological examination, treatment with preservative-free artificial tears were intensified to eight times daily or more, as tolerated, and treatment with steroid eye drops at four times daily was extended for more than 7 days, as appropriate.

Other toxicities included grade 2 infections in four patients, one patient had grade 3septic shock, and another patient grade 5COVID-19, whereas grade 3 thrombocytopenia was reported in two patients and grade 2 fatigue in four patients. One patient experienced a grade 2 infusion-related reaction during the first infusion of belantamab mafodotin, which resolved completely, and the patient received subsequent infusions without any complications. Dose reductions were reported in two patients due to thrombocytopenia. Overall, dose delays were reported in 19 patients (including both ocular and non-ocular toxicities). All patients continued with belantamab mafodotin administration every three weeks upon toxicity resolution. Two patients continued with monthly drug infusions after completing one year of treatment.

## 3. Discussion

Patients who are refractory to three classes of anti-myeloma drugs including at least one PI, one IMiD, and one anti-CD38 monoclonal antibody have poor prognosis with an estimated median OS less than a year [13]. In our study, belantamab mafodotin showed important efficacy and safety comparable to the results of the DREAMM-2 clinical trial [5,6] (Table 2). All 27 patients in our cohort had triple-class refractory MM and were heavily pretreated with a median of five prior lines of therapy. Importantly, the majority were refractory or had been exposed to carfilzomib and pomalidomide. The survival outcomes were more favorable for the responders, most of whom had sustained disease control and long-term remission.

Accumulating evidence from real-world studies is in accordance with our results (Table 2) [14,15,16,17,18,19]. A multicenter Italian study had a comparable cohort size (*n* = 28) to our study and reported an ORR of 40% and comparable survival outcomes [15]. In France, the IFM reported the outcomes of the compassionate use program of belantamab mafodotin including 97 patients with a median of five prior lines of therapies [17]. However, only 56% of the patients were triple-class refractory in this cohort. The median PFS and OS were 3.2 and 9.5 months, respectively. Another report from French centers participating in the ALFA study was presented at the last ASH meeting [20]. It included data from 184 patients and confirmed the above-mentioned results. The Israeli cohort included 106 patients with relapsed/refractory MM, 73% of whom were triple-class refractory [16]. Almost half of the patients (46%) showed response to treatment, whereas the median PFS was 4.7 months and the median OS was 14.5 months. The Spanish expanded access program included 156 patients with relapsed/refractory myeloma, including 88% with triple-class refractory disease [14,19]. Belantamab mafodotin resulted in an ORR of 42% and a median PFS and OS of 3.6 months and 11 months, respectively. Similar to our study, responders had significantly prolonged and sustained PFS and OS compared to non-responders. In both IFM and Israeli cohorts, survival outcomes did not differ between triple-class refractory and non-triple-class refractory patients. However, triple-class refractory patients had inferior PFS compared to others in the Spanish cohort, although this was not translated into a significant OS benefit.

The Mayo Clinic cohort included 36 patients with triple-class refractory MM and a median of eight prior lines of therapy [18]. One third of the patients responded to treatment, whereas the median PFS was 2 months and the median OS was 6.5 months. Comparable results have been also reported from three other real-world studies originating from the USA that included 137 [21], 82 [22], and 39 [23] patients, respectively, with a reported median PFS ranging from 1.8 months to 5 months and keratopathy rates ranging from 40% to 76%. However, these three studies have been published only as conference abstracts so far.

Approximately one third of our patients experienced keratopathy due to belantamab mafodotin administration. Reported rates of keratopathy vary slightly among real-world studies and the DREAMM-2 clinical trial range is from 32% to 72% [5,6,14,15,16,17,18]. This may be attributed to differences in the clinical practice and ophthalmological expertise in different clinical settings, as well as the robustness of adverse event reporting between clinical trials and real-world studies [9]. Ocular adverse events are the hallmark of belantamab mafodotin-related toxicities. Keratopathy and decrease in best corrected visual acuity (BCVA) may lead to treatment delays or even discontinuation in up to one fifth of the patients [14,15,16,17,18,19]. It seems that the rate of treatment discontinuation due to keratopathy is greater in real-world clinical practice than the DREAMM-2 study, in which only three patients discontinued due to corneal events [6,24]. Available data show that an extended time interval between subsequent doses and appropriate dose reduction from 2.5 mg/kg to 1.9 mg/kg may prevent the re-occurrence of corneal toxicity [24,25]. This is particularly important for patients who show response to belantamab mafodotin and develop corneal toxicity. Microcystic keratopathy due to monomethyl auristatin F is reversible, and, therefore, treatment discontinuation should be considered only in severe cases according to the available recommendations for the management of ocular toxicity due to belantamab mafodotin [26,27]. However, mitigating measures and a multidisciplinary team including the oncologist/hematologist, the ophthalmologist, and the nurse/physician assistant are essential for every patient with MM who receives treatment with belantamab mafodotin [26]. It must be noted that ocular adverse events may be attributed to other causes apart from belantamab mafodotin; in this context, a baseline ophthalmological assessment is important.

Other common toxicities with belantamab mafodotin include thrombocytopenia and infections. Thrombocytopenia is managed with supportive measures and dose delays and modifications [28]. Infection prevention is important and includes both immunization with vaccines and prophylactic use of antivirals and antibiotics [29]. Patients who receive anti-BCMA treatments are more likely to mount suboptimal humoral responses to vaccination due to defective B-cell immunity and are more prone to severe infections [30,31,32] However, T-cell-engaging bispecific antibodies seem to have a greater risk for infectious complications compared with belantamab mafodotin, and this should be considered during tailored treatment selection [33]. All patients with myeloma who receive B-cell-depleting therapies should be considered for prophylactic administration of immunoglobulins intravenously or subcutaneously, especially those with recurrent infections and/or serum IgG levels below 400 mg/dl [29]. Vaccination against common viral and bacterial pathogens and antibiotic prophylaxis is also important [29].

## 4. Materials and Methods

The patients were administered belantamab mafodotin monotherapy in the Greek named patient program (NPP). The patients started treatment from July 2018 to June 2021 in the NPP, and those who benefited from therapy continued treatment with commercial drugs thereafter. Belantamab mafodotin was indicated as monotherapy for the treatment of multiple myeloma in adult patients who had received at least four prior lines of therapy, whose disease was refractory to at least one PI, one IMiD, and one anti-CD38 monoclonal antibody, and who had demonstrated disease progression on the last therapy. The recommended dose was 2.5 mg/kg of belantamab mafodotin administered as an intravenous infusion once every 3 weeks until disease progression, the start of a new anti-myeloma treatment, consent withdrawal, or death. The study was conducted in accordance with the Declaration of Helsinki and informed consent was obtained from all subjects involved in the study.

In order to be eligible for the study, patients with triple-class refractory MM needed to have adequate organ system function, defined as absolute neutrophil count of at least 1.0 × 10^9^/L, hemoglobin at least 7 g/dL, platelet count of at least 50 × 10^9^/L, total bilirubin less than 1.5 times the upper limit of normal, aspartate aminotransferase less than 2.5 times the upper limit of normal, and estimated glomerular filtration rate by the Modification of Diet in Renal Disease (MDRD) equation of at least 30 mL/min/1.73 m^2^. Key exclusion criteria included evidence of active bleeding requiring intervention within the last four weeks, current corneal epithelial disease except for mild punctuate keratopathy, and active infection including hepatitis B and C.

An ophthalmologist examined each patient at baseline and before each drug administration in order to assess for any ocular toxicities. Prophylactic measures were taken to mitigate the risk for corneal toxicity associated with belantamab mafodotin. All patients were receiving preservative-free artificial tears at least four times daily beginning from cycle 1 day 1 until the end of treatment. Patients were also receiving steroid eye drops consisting of prednisolone phosphate 1%, dexamethasone 0.1%, or equivalent beginning from one day prior to each dose of belantamab mafodotin infusion and continuing for a total of seven consecutive days. Furthermore, patients were advised to apply a cooling eye mask during belantamab mafodotin infusion for up to four hours, as tolerated.

The primary efficacy study endpoint was ORR, and the secondary study efficacy endpoints were PFS, OS, and DCR, whereas the secondary safety endpoints included rate of adverse events and rate of ocular adverse events.

Descriptive statistics and non-parametric tests were used for baseline patient characteristics, whereas time to events was determined with the Kaplan–Meier method and subgroup differences were compared with the two-sided log rank test. All *p* values were two-sided and confidence intervals refer to 95% boundaries. Statistical analyses were performed with SPSS v.24 statistical software.

## 5. Conclusions

In conclusion, belantamab mafodotin showed a safety and efficacy profile consistent with the results of the DREAMM-2 study in triple-class refractory patients with MM in terms of survival benefit and tolerability in real-world practice. It seems that heavily pretreated patients who are responding to this regimen derive substantial survival benefit. Determining predictive factors for response to belantamab mafodotin in future studies will enable us to tailor our treatment approach to this challenging patient population.

## Figures and Tables

**Table 1:** Baseline patient characteristics.

Variables	*n* = 27
Age (median, range)	65 (41–81)
Male (*n*, %)	14 (52)
White (*n*, %)	27 (100)
Hemoglobin (mean, SD, g/dL)	10.8 (1.7)
Creatinine (mean, SD, mg/dL)	0.98 (0.4)
Abnormal LDH (*n*, %)	16 (59)
Prior lines of therapy (median, range)	5 (4–10)
Prior bortezomib (*n*, %)	27 (100)
Prior lenalidomide (*n*, %)	27 (100)
Prior anti-CD38 mAb (*n*, %)	27 (100)
Prior alkylator (*n*, %)	25 (93)
Prior carfilzomib (*n*, %)	24 (89)
Prior pomalidomide (*n*, %)	19 (70)
Only biochemical relapse (*n*, %)	16 (59)
EMP relapse (*n*, %)	3 (11.1)
ISS stage 1 (*n*, %)	9 (33)
ISS stage 2 (*n*, %)	13 (48)
ISS stage 3 (*n*, %)	5 (19)
R-ISS stage 1 * (*n*, %)	2 (14)
R-ISS stage 2 * (*n*, %)	10 (72)
R-ISS stage 3 * (*n*, %)	2 (14)
IgGκ myeloma (*n*, %)	9 (33.3)
IgGλ myeloma (*n*, %)	4 (14.8)
IgAκ myeloma (*n*, %)	3 (11.1)
IgAλ myeloma (*n*, %)	1 (3.7)
κLC-only myeloma (*n*, %)	5 (18.5)
λLC-only myeloma (*n*, %)	5 (18.5)

* Data available for 14 patients. SD: standard deviation; LDH: lactate dehydrogenase; mAb: monoclonal antibody; EMP: extramedullary plasmacytoma; (R) ISS: (revised) international staging system; Ig: immunoglobulin; LC: light chain.

**Table 2:** Comparison among our cohort with the DREAMM-2 clinical trial and other real-world studies.

	Athens Cohort	DREAMM-2 Study	IFM 2020-04 Cohort	Mayo Clinic Cohort	Spanish Cohort	Italian Cohort	Israeli Cohort
*n*	27	97	97	36	156	28	106
Age (median, range)	65 (41–81)	65 (60–70)	66 (37–82)	61 (37–83)	73 (40–89)	68 (51–83)	69 (36–88)
Males (*n*, %)	14 (52)	51 (53)	49 (51)	23 (64)	82 (46)	16 (57)	60 (57)
Prior lines of therapy (median, range)	5 (4–10)	7 (3–21)	5 (3–12)	8 (7–11)	5 (1–10)	6 (3–14)	6 (2–11)
ISS I/II/III (%)	33/48/19	22/34/43	36/39/25	25/17/33	29/31/33	NR	43/30/26
High-risk cytogenetics (*n*, %)	6/15 (40)	41 (42)	27/66 (41)	14 (41)	*	NR	27 (43)
Triple-class refractory (*n*, %)	27 (100)	97 (100)	55 (56)	36 (100)	125 (80)	28 (100)	77 (73)
Prior ASCT (*n*, %)	25 (93)	73 (75)	70 (72)	27 (75)	101 (65)	20 (71)	62 (59)
Prior carfilzomib (*n*, %)	24 (89)	74 (76)	11 (11)	36 (100)	NR	24 (86)	77 (73)
Prior pomalidomide (*n*, %)	19 (70)	89 (92)	60 (62)	36 (100)	NR	NR	82 (77)
Median PFS (months)	2	2.8	3.2	2	3.6	3	4.7
Landmark median OS (months)	16	13.7	9.5	6.5	11	8	14.5
ORR (*n*, %)	14 (52)	31 (32)	37 (38)	12 (33)	14 (42)	11 (40)	46 (46)
sCR/CR (*n*, %)	3 (11)	7 (7)	8 (8)	2 (6)	4 (12)	3 (11)	4 (4)
VGPR (*n*, %)	5 (19)	11 (11)	11 (11)	3 (8)	2 (6)	3 (11)	14 (14)
PR (*n*, %)	6 (22)	13 (13)	18 (19)	7 (19)	8 (24)	5 (18)	28 (28)
Keratopathy (*n*, %)	9 (33)	68 (72)	39 (38)	15 (43)	73 (88) **	9 (32)	65 (68)
IRR (*n*, %)	1 (4)	20 (21)	10 (10)	2 (5)	NR	0	8 (8)

* *n* = 17 with del17p, *n* = 15 with t(4;14), *n* = 28 with 1q21+, *n* = 1 with t(14;20). ** The incidence of ocular toxicity was reported. ISS: international staging system; ASCT: autologous stem cell transplant; PFS: progression-free survival; OS: overall survival; ORR: overall response rate; (s)CR: (stringent) complete response; VGPR: very good partial response; PR: partial response; IRR: infusion-related reaction; NR: not reported.

## Data Availability

Data are available upon reasonable request from the corresponding author.
